# External Quality of Mandarins: Influence of Fruit Appearance Characteristics on Consumer Choice

**DOI:** 10.3390/foods10092188

**Published:** 2021-09-15

**Authors:** Paula Tarancón, Amparo Tárrega, Mónica González, Cristina Besada

**Affiliations:** 1 Centro de Tecnología Postcosecha, Valencian Institute for Agricultural Research (IVIA), Carretera Moncada-Náquera, kilometer 4.5, 46113 Moncada, Valencia, Spain; tarancon_pau@gva.es; 2 Instituto de Agroquímica y Tecnología de Alimentos (IATA-CSIC), Agustín Escardino 7, 46980 Paterna, Valencia, Spain; atarrega@iata.csic.es (A.T.); moni.gonzalez@iata.csic.es (M.G.)

**Keywords:** choice-based conjoint analysis, rind colour, calyx, rind turgor, leaf, waxing, coating

## Abstract

In a pre-purchase situation, consumer perception of mandarin quality is almost exclusively based on fruit appearance. Determination of consumer requirements in this regard is needed to preserve the current success of this crop in markets worldwide. In this study, the effect on consumer choice of different appearance characteristics that can occur mainly in early-season mandarins was quantified. Two independent Choice-Based Conjoint Analyses were performed to evaluate the effect of different external mandarin factors: (1) two factors linked mainly with harvesting practices: ‘Leaf’ (no leaves but sound calyx/fresh leaf/dehydrated leaf) and ‘Rind Colour’ (orange colour/rind with slightly greenish areas); (2) three factors related to postharvest handling: ‘Calyx Condition’ (sound/blackening/detached),’Waxing’ (absence/presence of wax), and ‘Rind Condition’ (dehydrated/turgid). A total of 280 consumers participated in each study. The evaluation of the factors linked with harvesting revealed four different choice patterns. Leaf presence was appreciated only by a small group of consumers (20%), and the presence of greenish areas on the rind was perceived mostly as a negative characteristic. Among the evaluated postharvest-related factors, ‘Waxing’ and ‘Calyx condition’ had the strongest effect on consumer choice. Consumers showed marked preferences for mandarins that had been waxed and presented shine and gloss. Calyx blackening and detachment had a negative effect mainly on waxed fruit, while rind dehydration more negatively affected the fruit that had not been waxed. Consumer perception of mandarin quality is importantly affected by external mandarin characteristics. The information herein reported can be very useful for the citrus industry for adapting quality control criteria that respond to consumer demands.

## 1. Introduction

The global citrus fruit production has been estimated to be close to 158 million tons, and represents the major fruit tree crop in the world [1]. Of the main citrus species, the production of oranges is the most important in terms of quantity. However, while the world orange production increased by 15% between 2009 and 2019, mandarin production rose by over 60% during the same period [1]. Hence, mandarin fruit, with its convenient size and easy peeling feature, is gaining market ground compared to oranges.

In order to maintain and enhance the current success of mandarins in markets around the world, it is necessary for all supply chain stakeholders (producers, packing houses, marketers, and distributors) to be aware of how to meet consumer quality requirements.

Flavour, taste, texture, and internal colour are intrinsic characteristics to mandarins that have been reported to influence consumer preferences [2,3,4,5]. However, in a pre-purchase situation, not all these quality attributes are obvious and consumers have to base their choice almost exclusively on external appearance. 

Commercial standards on this regard have been traditionally based on technical experts’ criteria. However, as stated by Kader [6], this implies the risk of creating a gap with respect to consumer expectations, with the consequent commercial problems. 

The influence of aspects such as price, colour, size, seediness, skin blemishes, production region label, and organic production has been evaluated by Campbell et al. [7] in varieties from the satsumas group. This study, in which mandarin images that combined different factors were evaluated by means of a purchase intention scale, reported that consumers generally showed preferences for the biggest, unseeded, and non-blemished fruit. Moreover, these authors found that mandarins with yellow-orange skin colouration were more frequently chosen than those with orange skin. More recently, Morales et al. [8] evaluated the effect on consumer preferences of enhancing citrus fruit colouration by applying degreening treatment, and found that consumers preferred the most coloured mandarins with completely orange skin, which disagrees with that previously reported by Campbell et al. [7]. 

Moreover, in the last few years, some distributors have started commercialising early-season mandarins with still greenish rind areas as a sign of freshness linked with a recent harvest or to a sense of being more “natural” (as no postharvest degreening treatment is applied). In line with enhancing consumer perception of freshness and naturalness linked with local food, the commercialisation of mandarins with leaves is increasing. In the same line, different studies have reported consumer voicing more concerns about the use of fruit waxes [9,10], and the commercialisation of mandarins with no added wax has grown in recent years in association with increased organic farming.

Recently, Tarancón et al. [4] confirmed the negative impact of skin damage on consumer preferences previously described by Campbell et al. [7]. In the aforementioned study [4], skin disorders associated with chilling injury manifestation during cold storage led to a significant decrease in consumer preferences and purchase intention in relation to harvest time. However, other mandarin appearance characteristics linked with postharvest handling are likely to affect the consumer perception of quality, but have not yet been evaluated in this regard. Thus, degreening treatment applied at early season mandarins to enhance rind coloration may lead to the manifestation of certain external disorders. A collateral effect of ethylene degreening treatment is that too high or too prolonged ethylene exposure may result in calyx blackening, or even calyx detachment, which would clearly affect fruit appearance [11,12]. 

Therefore, while there is no doubt about the positive effect of certain factors such as size (larger fruit), seedlessness, and absence of blemishes on consumer mandarin choice, a gap in information about the impact of factors such as leaf presence, wax absence and calyx condition still exists. Moreover, there is still some controversy about the influence of rind colouration. Besides scientific interest in obtaining this information, it is crucial to set quality controls that ensure that consumer requirements are met.

In this context, our study aimed to bridge this gap by quantifying the effect on consumer mandarin preferences of different external appearance factors that are associated with harvesting (Rind Colour and Leaf Presence) and postharvest (Calyx Condition, Waxing and Rind Condition) practices. To this end, we used the choice-based conjoint analysis, which allowed us to determine the impact of different factor combinations and to identify consumer segments based on their choice profiles.

## 2. Materials and Methods

### 2.1. Conjoint Analysis

There are two main types of conjoint analysis: rating-based conjoint, which is based on consumer evaluations of products individually using hedonic or willingness to buy scales, and choice-based conjoint (CBC), which relies on the selection of the preferred product among several alternatives [13]. The CBC analysis has been reported to be more similar to real market behaviour than rating-based conjoint [13,14]. Subjects are asked to choose the alternative that they prefer from a small set of profiles, which is a similar form of decision making to those that consumer make in their everyday lives.

Based on these considerations, the CBC analysis was used in this study to examine the influence of different mandarin appearance factors, and their combination, on consumer choice.

### 2.2. Conjoint Study Design and Procedure

Mandarins cv. Clemenules are one of the most important early-season mandarin variety in Spain. Therefore, this variety was selected as representative of early-season varieties. Fruits were collected from a packing house in Valencia (Spain), and whenever necessary, they underwent different postharvest conditions (storage, waxing) to obtain the external aspect required for evaluation.

Two independent choice-based conjoint analyses were performed: one to evaluate the effect of harvesting-related factors and another to evaluate the effect of postharvest handling-related factors. 

The two harvesting-related factors and their levels were: ‘Leaf’ (no leaves but sound calyx/fresh leaf/dehydrated leaf); ‘Rind colour’ (orange/small slightly greenish areas) (all the samples were waxed and had a turgid rind) (Table 1). The three postharvest handling-related factors and the evaluated levels were: ‘Calyx Condition’ (sound/detached/blackened); ‘Waxing’—wax application for shine (Yes/No); ‘Rind Condition’ (turgid/dehydrated) (all the samples had an orange-coloured rind) (Table 2). 

In both cases, the “sound calyx-wax-orange rind-turgid rind” combination was considered the “standard mandarin” as it is the most widely commercialised one. Thus, the effect of the different attribute levels on consumer choice was studied comparatively to the standard mandarin choice. The two factors associated with harvesting practices, ‘Leaf’ and ‘Rind Colour’, were initially hypothesised to have a positive effect on consumer choice, as we expected consumers to associate the presence of both fresh leaves and slightly greenish areas on the rind with freshness. The three postharvest-related factors were hypothesised to have a negative effect on consumer choice as we evaluated the effect of detached/blackened calyx, absence of wax, and dehydrated rind compared to the standard mandarin. 

High-quality colour images of mandarins displaying the previously explained combination of factors (Table 1 and Table 2) were taken by a professional photographer (Figures S1 and S2). Six images of mandarin combining the two harvesting factors (3 × 2 full factorial design) and twelve images of mandarin combining the three postharvest factors (3 × 2 × 2, full factorial design) were obtained.

Consumer evaluations were made separately for conjoint study 1 (harvesting factors) and conjoint study 2 (postharvest factors). Consumers completed an online questionnaire that was implemented using Compusense Cloud (Compusense, Guelph, ON, Canada). 

A total of 280 Spanish consumers (60% female, 40% male) participated in study 1 and 280 consumers (59% female, 41% male) in study 2. The participants took part in only one of the two studies. In each study, participants were presented with eight choice sets, each composed of three mandarin images. For each choice set, consumers were asked to observe the mandarin images and to indicate the mandarin that they would choose to eat. An incomplete block design was employed to generate the eight comparison sets to be presented to the different participants. XLSTAT software was used to this end.

After evaluating the different images, participants answered some demographic questions, such as their consumption frequency, gender, and age.

### 2.3. Statistical Analysis

Analysis of choice data was made by multinomial logit model regression using the XLSTAT statistical software. The model included the main effects (i.e., individual attributes) and binary interaction between them. This enabled a deeper understanding of how the experimental factors determined consumer responses. The utility value for each level or interaction was obtained. In the analysis, one of the levels of each factor was considered as reference. 

Additionally, in conjoint study 1, individual utilities were obtained for each consumer by logit model regression considering only the main effects. A hierarchical cluster analysis (HCA) was applied to the individual utilities to identify groups of consumers who had similar choice patterns. Euclidean distances and Ward’s aggregation method were considered. 

## 3. Results

### 3.1. Effect of Attributes Associated with Harvesting on Consumer Choice

‘Rind colour’ (orange rind/small slightly greenish areas) and ‘Leaf’ (sound calyx/fresh leaf/dehydrated leaf) were the fruit harvesting-related attributes evaluated in this study. The results were expressed as the impact of the different factors on consumer choice compared to the “standard mandarin” choice (sound calyx, orange rind), which was equalled to zero to this end. The statistics for the likelihood ratio and Score and Wald indicated that the model was not significant (*p* > 0.05). Accordingly, neither the two factors, nor their interaction had a significant effect on consumer choice (data not shown). In view of this result, hierarchical cluster analysis was performed to gain a better understanding of consumer mandarin choice. To this end, the utilities for each factor level were obtained for each participant and a cluster analysis was applied.

Four different choice profiles were identified among consumers. As shown in Table S1, the studied factors were significant for two identified segments of participants (Cluster 2 with 38% of the participants, and Cluster 3 with 13%), but not for the consumers in Clusters 1 and 4. This result shows the existence of different choice profiles among the identified groups. 

The values of the utilities and significance for the harvesting-related factors for each cluster are depicted in Table 3. The consumers in Cluster 3 were the only ones for whom leaf presence had a positive and significant impact on their choice, irrespectively of leaf aspect. Leaf presence had an opposite effect on the preferences of the Cluster 2 consumers as consumer choice was negatively affected irrespectively of leaf condition. The choice of this consumer group (Cluster 2) was also determined by colour rind, as the presence of greenish areas had a negative effect. For the consumers in Clusters 1 and 4, the impact of the factors on choice was not significant. The utilities of Cluster 1 were very low, while those of Cluster 4 were higher. In both cases, utilities generally obtained negative values, which indicates that the evaluated factors tended to negatively impact consumer choice.

**Figure 1:**
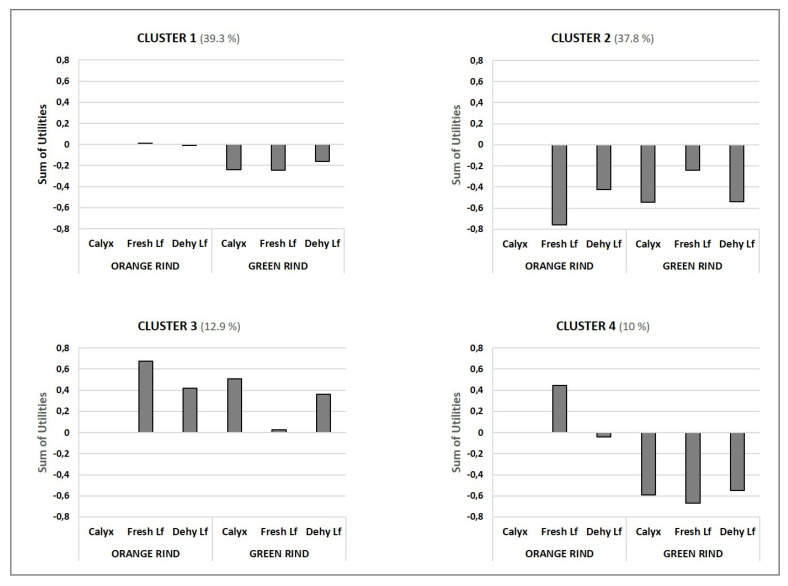
Global effect (sum of utilities of the individual factors and their interactions) of harvesting factor combinations on consumer mandarin choice. The total number of participants in the conjoint test was 280. The results are expressed in relation to the “standard mandarin” (Sound calyx, Orange rind). All the fruit were waxed and had a turgid rind. Lf: leaf.

The global effect of the two factor combinations on consumer choice, compared to the “standard mandarin”, is shown in Figure 1. The consumers in Cluster 1 (39.3% of the participants) showed preferences for orange-coloured mandarins, and leaf presence had no effect. The presence of greenish areas on the rind negatively affected their choice, and the irrelevance of leaf presence was confirmed.

In total, 38% of consumers (Cluster 2) preferred the “standard mandarin”, i.e., orange-coloured fruit with a sound calyx. For this consumer segment, the presence of either leaf or greenish areas on the rind negatively impacted their choice. 

A very different pattern was detected in Cluster 3 (13% of the participants), which grouped those participants for whom the presence of both leaf and greenish areas on the rind had a positive effect. It is worth mentioning that while fresh leaf presence was positive when fruit was orange-coloured, this effect was almost imperceptible in the fruit with rind areas that were slightly green. 

Finally, 10% of the consumers included in Cluster 4 were characterised by the negative effect that green colour had on their mandarin selection decision making. For this group, leaf state was decisive, but only when choosing orange-coloured mandarins, when fresh leaf had a positive impact, while dehydrated leaf barely affected consumers’ decision.

An analysis of the demographic characteristics of the participants in each cluster was performed by means of the z-test for multiple proportions (data not shown). This analysis revealed no significant effect for age, gender, or consumption frequency (*p* > 0.05). This indicates that the four identified consumers’ choice patterns were not linked with demographic factors. 

### 3.2. Effect of Attributes Associated with Postharvest Handling on Consumer Choice

The group of attributes directly related to postharvest handling included ‘Waxing’, ‘Rind condition’, and ‘Calyx condition’, which were analysed together. As in the evaluation of the harvesting factors, the “standard mandarin” choice (sound calyx, wax, turgent rind) was equalled to zero. In this case, the chi-squared estimated values for the likelihood ratio, and Score and Wald statistics indicated that the model was highly significant (*p* < 0.001). Accordingly, the study conducted in detail of the effect of the different factors revealed that the three individual attributes significantly affected consumers’ choice of their preferred mandarin (Table S2). An effect of the ‘Waxing’–‘Calyx condition’ and ‘Calyx condition’–‘Rind condition’ interactions were also detected (*p* < 0.05). 

According to our initial hypothesis, all the evaluated attributes levels (detached and blackened calyx, absence of wax, dehydrated rind) had a negative impact on consumer perception of quality and, consequently, in their choice, as indicated by the negative values of their utilities (Table 4). Regarding the interaction between factors, the positive values of utilities displayed by four of the significant interactions indicated that the impact of combining two of the factors was not as negative as expected by adding the impacts of the individual factors. 

**Figure 2:**
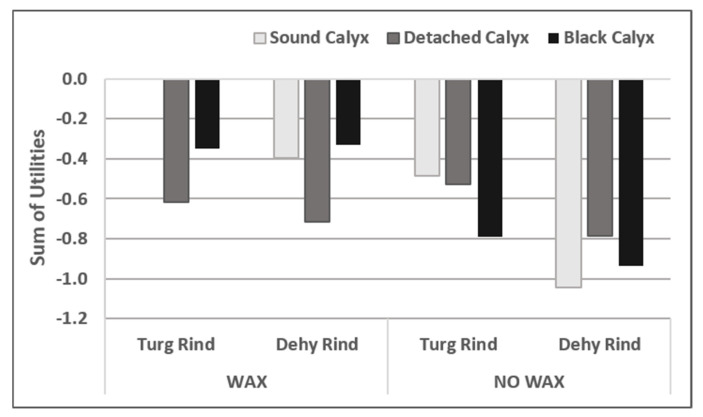
Global effect (sum of utilities of individual factors and their interactions) of combinations of postharvest-related factors on consumer mandarin choice. The total number of participants in the conjoint test was 280. The results are expressed in relation to “standard mandarin” (Sound calyx, Wax, Turgid rind). All the fruit had an orange-coloured rind. Dehy: dehydrated; Turg: Turgid.

As observed in Figure 2, the absence of waxing had a negative impact on consumer mandarin selection, and this effect was more marked for the fruit that had a dehydrated rind than for those with a turgid rind. It was noteworthy that the negative impact of no waxing was stronger in those fruit with a sound or black calyx than in those that had lost the calyx. 

Of the waxed samples, rind dehydration negatively affected the selection of the fruit with calyx, but had no effect on the fruit with a black or detached calyx. However, consumer selection among the non-waxed mandarins was negatively affected by rind dehydration irrespectively of Calyx Condition. The non-waxed fruit with a dehydrated rind were the least likely to be chosen by consumers. 

Finally, consumer choice was more negatively affected by the absence of calyx than by calyx blackening on waxed fruit. However, of the non-waxed fruit, consumers preferred fruit with no calyx over blackened-calyx fruit.

To summarise, of the evaluated postharvest factors, calyx detachment most negatively affected consumer choice, mainly when they had to select among waxed fruit. Absence of wax was the second most impacting factor, of which the negative impact was observed mainly when consumers had to choose among mandarins with a sound or black calyx. 

## 4. Discussion

The five appearance attributes herein evaluated were divided into harvesting-related attributes (Leaf and Rind Colour) and postharvest handling-related attributes (Calyx Condition, Waxing, and Rind Condition), and two individual conjoint analyses were performed. The division of the factors into these two groups was motivated mainly by our initial hypothesis: compared to the “standard mandarin”, the harvesting-related attributes (leaf presence and rind with slightly greenish areas) were expected to have a positive effect in choice linked to a higher quality perception due to perceived freshness, while the evaluated postharvest attributes (blackened/detached calyx, no waxing, and dehydrated rind) were expected to negatively affect consumer choice. About the harvesting-related factors, it is important to explain one point. In this study, ‘Rind colour’ was taken as a harvesting-related factor because early varieties usually reach internal maturity while the skin is still green in citrus-growing areas with tropical and subtropical climate conditions [15]. Thus, rind colour may be the limiting factor for harvesting. However, in most citrus-producing regions, the fruit that reach internal maturity is usually harvested with insufficient external colour and then submitted to degreening treatments, which stimulates chlorophyll degradation and carotenoids biosynthesis [11]. In these cases, the desirable rind colour for commercialisation is obtained by postharvest treatments.

The conjoint analysis, which was performed by taking into account the harvesting factors data from all the participants, showed a very low impact for ‘Leaf’ and ‘Rind Colour’ on consumer choice, which was an unexpected result. Therefore, we studied the data evaluation in-depth by performing HCA, as previously described by Lee et al. [16]. This methodology allowed us to identify four consumer choice profiles. Surprisingly, our initial hypothesis was true, but only for a relatively small number of consumers (13%, Cluster 3), for whom the two attributes under study had a positive effect on their mandarin selection. This may indicate a trend in this regard, but currently, only a small group of consumers prefer mandarins with leaf and green tones on the rind. 

When the utilities of the individual factors and their interactions were added to analyse the effect of the factor combinations, for most participants (Clusters 1, 2, and 4), the presence of greenish areas on the rind negatively affected their mandarin choices. This result disagrees with Campbell et al. [7], but confirms what was reported by Morales et al. [8], who found that consumers showed preferences for citrus fruit (mandarins and oranges) with homogeneous orange-coloured rinds. According to these authors, the presence of greenish areas on the rind results in low-quality expectations due to their immaturity.

Freshness has been reported to be one of the main drivers for consumer choice of different fruit, including mandarins [9,17], and appearance plays a significant role in consumer perception of fruit freshness [18]. In line with this, the citrus industry has opted to commercialise mandarins with leaf presence in recent years. This commercial strategy is based on the assumption that consumers associate leaf presence with fruit that has been recently picked. However, our results showed that for most participants, leaf presence had no positive effect when they selected mandarins. This is a very interesting result because handling citrus fruit with leaf presence implies a significant additional cost for the citrus industry. As the decision choice of food products can be affected by nationality [19,20,21], our finding opens the door to new studies with a view to elucidate whether this pattern is also true for other international markets. 

Moreover, for the 20% of consumers for whom leaf presence was a positive factor (Clusters 3 and 4), leaf state was also a determinant. Thus, leaf freshness was crucial for the consumers in Cluster 4 when selecting among orange-coloured mandarins. A noteworthy pattern was also detected for the consumers in Cluster 3. This group of consumers preferred mandarins with fresh leaf presence compared to those with dehydrated leaves among orange-coloured fruit, which is probably related to their perception of freshness. However, for the mandarins with greenish rind areas, those with fresh leaf presence were less appreciated. Our hypothesis is that for this group of consumers, fresh leaf presence may enhance their expectations of immature fruit linked with greenish areas on the rind. 

With regard to the postharvest-related factors herein evaluated, our results confirmed our initial hypothesis of them having a negative effect on perceived quality. Thus, the “standard mandarin” was that preferred by consumers. 

The ‘Calyx Condition’ and ‘Waxing’ factors had a strong impact on consumer choice. Waxing fruit is a long-standing practice that helps to prolong the shelf life of fruit [22,23,24]. Moreover, adding certain components such as shellac to waxes makes fruit shiny. In this study, fruit shine and gloss were the external characteristics that mandarins acquired after waxing. Our results confirmed that they were greatly appreciated by consumers. 

However, the study conducted by Cliff et al. [10], in which consumers were interviewed about their perception of fruit waxing, reported that this kind of technology is becoming negatively perceived by consumers. In the present study, consumers did not receive any information about fruit other than appearance. Therefore, doubts arise as to whether the participants associated, or did not, the shine and gloss of waxed mandarins with wax addition. This would be an interesting subject for future research. 

The CBC design used in this study allowed us to visualise the effect of combining different factors on consumer decision, which coincides with the proposal of Yu et al. [25]. These authors affirmed that with the choice of experimental design, incorporating attribute interaction effects into both the design and model estimation stages is recommended for precise predictions.

After evaluating the effect of the factor combinations, we can affirm that the least attractive mandarins for consumers were characterised by having no shine in combination with dehydrated rind. It was also interesting that calyx state had an effect when consumers evaluated waxed fruit, when mandarins with a blackened calyx were preferred to those with no calyx. However, as the general fruit aspect worsened (no waxed fruit with dehydrated rind), the impact of calyx on consumer choice became secondary.

This study quantifies the impact of different factors on consumer choice for the first time, which is valuable information for the citrus industry. However, some limitations of this study should be mentioned. In the choice task presented herein, consumers were shown images of individual fruit. This helps consumers to pay attention to the factors being evaluated, and we assume that they made their decisions based on them. However, in a real purchase situation, consumers have to base their decisions on the appearance of a fruit lot, and not only on one. Therefore, certain variability among fruit as regards the evaluated factors is expected. A clear example of this is the calyx condition because it may differ among fruit, even if they have undergone the same postharvest treatments. Determining the minimum percentage of the fruit showing a specific characteristic in a commercial lot that leads to changes in consumer decisions would be an interesting topic for future studies. Such information would supplement the results herein reported and would help to optimise quality controls. In the same line, the use of observational techniques, such as eye-tracking technology to investigate consumers’ visual behaviour, would be very useful for determining to what extent fruit characteristics capture consumers’ attention, and our group is already working on this.

## 5. Conclusions

The evaluation of the effect of harvesting practices-related factors (Leaf and Rind Colour) revealed four different consumer segments based on their choice profiles. Most consumers (Clusters 1 and 2) indicated preferences for the “standard mandarin” (sound calyx, orange rind), and the presence of greenish areas on the rind negatively impacted their choice. The main difference between these two consumer segments was that leaf presence had no effect on Cluster 1 consumers, but it negatively affected the choice of the Cluster 2 consumers. Leaf presence was only appreciated by 23% of the participants (Clusters 3 and 4), mainly on orange-coloured fruit. The Cluster 3 consumers (13%) were the only ones for whom greenish areas on the rind had a positive impact.

A marked effect on consumer choice was also detected when the appearance factors linked to postharvest handling were evaluated. In this case, consumers’ response was more homogeneous and a general pattern was identified. The shine and gloss that waxing confers mandarins markedly and positively affected consumer mandarin selection. The extent of calyx deterioration (blackening, detachment) was a determinant for consumer selection for waxed fruit, while rind turgor was more relevant for choosing non-waxed fruit. The information reported herein is very useful for the citrus industry in order to set quality standards that meet consumer requirements.

## Figures and Tables

**Table 1:** Levels of mandarin external aspects linked with harvesting factors evaluated in the conjoint design. All the samples were waxed and had a turgid rind.

Harvesting Factors	Level
Leaf	Sound Calyx ^S^
	Fresh
	Dehydrated
Colour	Orange ^S^
	Slight greenish areas

^S^ Indicates the level considered standard in each factor.

**Table 2:** Levels of mandarin external aspects linked with postharvest factors evaluated in the conjoint design. All the samples had an orange-coloured rind.

Postharvest Factors	Level
Calyx	Sound ^S^
	Detached
	Blackened
Waxing	Yes ^S^
	No
Rind	Turgid ^S^
	Dehydrated

^S^ Indicates the level considered standard in each factor.

**Table 3:** Values of utilities for the contribution of the harvesting-related factors and their interactions to consumer mandarin choice. The total number of participants in the conjoint test was 280. The percentages in brackets next to clusters indicate the proportion of consumers in each one. Utilities are expressed in relation to that of the standard mandarin (Sound calyx, Wax, Turgid rind). Those factors and their interactions that were significant (*p* < 0.05) according to the Ward test are indicated in bold.

Level	Utilities			
	Cluster 1 (39%)	Cluster 2 (38%)	Cluster 3 (13%)	Cluster 4 (10%)
Fresh leaf (vs. Sound calyx)	0.009 (0.964)	**−0.760 (<0.0001)**	0.678 **(0.009)**	0.443 (0.150)
Dehydrated leaf (vs. Sound calyx)	−0.009 (0.964)	**−0.425 (0.001)**	0.420 (0.116)	−0.042 (0.894)
Green rind (vs. Orange rind)	−0.239 (0.091)	**−0.545 (<0.0001)**	0.510 (0.054)	−0.590 (0.061)
Sound calyx × Green rind (vs. Sound calyx × Orange rind)	0.000	0.000	0.000	0.000
Fresh leaf × Orange rind (vs. Sound calyx × Orange rind)	0.000	0.000	0.000	0.000
Fresh leaf × Green rind (vs. Sound calyx × Orange rind)	−0.014 (0.948)	**1.065 (<0.0001)**	**−1.161 (0.003)**	−0.525 (0.282)
Dehydrated leaf × Orange rind (vs. Sound calyx × Orange rind)	0.000	0.000	0.000	0.000
Dehydrated leaf × Green rind (vs. Sound calyx × Orange rind)	0.090 (0.662)	**0.429 (0.038)**	−0.567 (0.134)	0.081 (0.853)

**Table 4:** Values of utilities for the contribution of the postharvest-related factors and their interactions to consumer mandarin choice. The total number of participants in the conjoint test was 280. Utilities are expressed in relation to that of the standard mandarin (Sound calyx, Wax, Turgid rind). Those factors and interactions that were significant (*p* < 0.05) according to the Ward test are indicated in bold.

Level	Utilities	Significance
Detached calyx (vs. Sound calyx)	**−0.617**	**<0.0001**
Black calyx (vs. Sound calyx)	**−0.348**	**0.001**
No wax (vs. Wax)	**−0.484**	**0.000**
Dehydrated rind (vs. Turgid rind)	**−0.395**	**0.007**
Sound calyx × No wax (vs. Sound calyx × Wax)	0.000	_ _ _
Black calyx × Wax (vs. Sound calyx × Wax)	0.000	_ _ _
Black calyx × No wax (vs. Sound calyx × Wax)	0.041	0.749
Detached calyx × Wax (vs. Sound calyx × Wax)	**0.000**	_ _ _
Detached calyx × No wax (vs. Sound calyx × Wax)	**0.575**	**<0.0001**
Sound calyx × Dehydrated rind (vs. Sound calyx × Turgid rind)	0.000	_ _ _
Black calyx × Turgid rind (vs. Sound calyx × Turgid rind)	0.000	_ _ _
Black calyx × Dehydrated rind (vs. Sound calyx × Turgid rind)	**0.416**	**0.005**
Detached calyx × Turgid rind (vs. Sound calyx × Turgid rind)	0.000	_ _ _
Detached calyx × Dehydrated rind (vs. Sound calyx × Turgid rind)	0.297	0.055
Wax × Dehydrated rind (vs. Wax × Turgid rind)	0.000	_ _ _
Not wax × Turgid rind (vs. Wax × Turgid rind)	0.000	_ _ _
Not wax × Dehydrated rind (vs. Wax × Turgid rind)	−0.162	0.242

## Data Availability

The data that support the findings of this study are available on request from the corresponding author.

## Supplementary Materials

The following are available online at https://www.mdpi.com/article/10.3390/foods10092188/s1, Figure S1: Images of mandarins used to evaluate the effect of harvesting-related factors on consumer choice. (A) Standard mandarin (sound calyx-orange rind); (B) fresh leaf-orange rind, (C) dehydrated leaf-orange rind, (D) sound calyx-small green areas, (E) fresh leaf-small green areas, (F) dehydrated leaf-small green areas. Figure S2: Images of mandarins used to evaluate the effect of postharvest-related factors on consumer choice. (A) Standard mandarin (sound calyx-wax-turgid rind), (B) sound calyx-no wax-turgid rind, (C) sound calyx-wax-dehydrated rind, (D) sound calyx-no wax-dehydrated rind, (E) detached calyx-wax-turgid rind, (F) detached calyx-no wax-turgid rind, (G) detached calyx-wax-dehydrated rind, (H) detached calyx-no wax-dehydrated rind, (I) black calyx-wax-turgid rind, (J) black calyx-no wax-turgid rind, (K) black calyx-wax-dehydrated rind, (L) black calyx-no wax-dehydrated rind. Table S1: Level of significance of the different harvesting-related factors and their interaction for the four identified Clusters. Table S2: Level of significance of the different postharvest-related factors and their interactions on the consumer mandarin choice. Cd: condition.
