# Supplementary material for: External Quality of Mandarins: Influence of Fruit Appearance Characteristics on Consumer Choice
Source: Foods. 2021 Sep 15;10(9):2188. doi: 10.3390/foods10092188 (PMC8466745; doi:10.3390/foods10092188)
Supplement: Supplementary file 1 [file foods-10-02188-s001.zip › foods-1357678 supp.pdf]

SUPPLEMENTARY MATERIAL

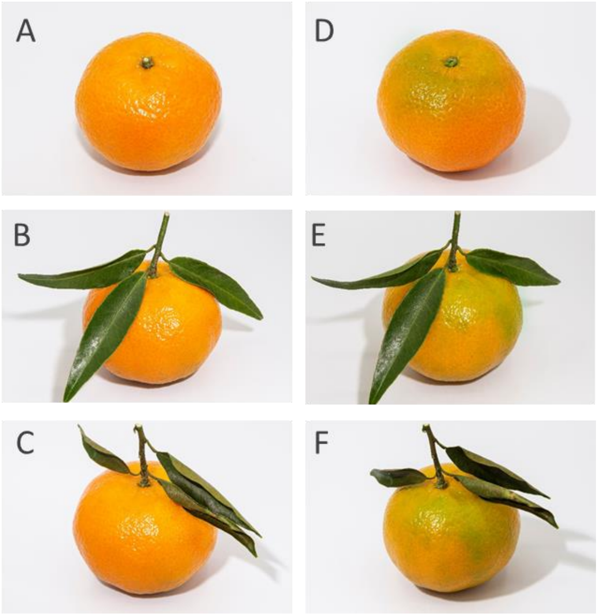

Figure S1. Images of mandarins used to evaluate the effect of Harvesting related factors on consumer choice. **A)** Standard mandarin (sound calyx-orange rind); **B)** fresh leaf-orange rind, **C)** dehydrated leaf-orange rind, **D)** sound calyx-small green areas, **E)** fresh leaf-small green areas, **F)** dehydrated leaf-small green areas. All mandarins were waxed.

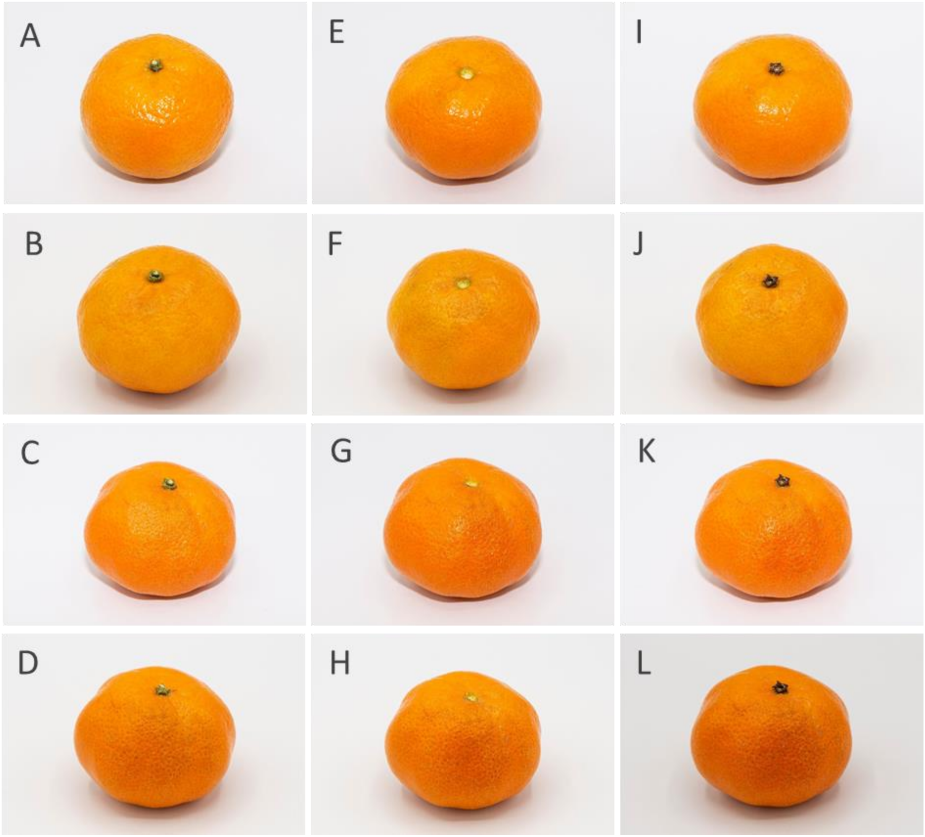

Figure S2. Images of mandarins used to evaluate the effect of Posharvest related factors on consumer choice. **A)** Standard mandarin (sound calyx-wax-turgid rind), **B)** sound calyx-no wax-turgid rind, **C)** sound calyx-wax-dehydrated rind, **D)** sound calyx-no wax-dehydrated rind, **E)** detached calyx-wax-turgid rind, **F)** detached calyx-no wax-turgid rind, **G)** detached calyx-wax-dehydrated rind, **H)** detached calyx-no wax-dehydrated rind, **I)** black calyx-wax-turgid rind, **J)** black calyx-no wax-turgid rind, **K)** black calyx-wax-dehydrated rind, **L)** black calyx-no wax-dehydrated rind.

Table S1. Level of significance of the different Harvesting related factors and their interaction for the four identified Clusters. The total number of participants in the conjoint test was 280. Percentages in brackets next to clusters indicates the proportion of consumers in each one.

HARVESTING FACTORS				
Attributes	***Cluster 1*** (39.3 %)	***Cluster 2*** (37.8 %)	***Cluster 3*** (12.9 %)	***Cluster 4*** (10 %)
Rind Colour	0.091	**<0.0001**	0.054	0.061
Leaf	0.991	**<0.0001**	**0.034**	0.179
Leaf * Rind Colour	0.841	**<0.0001**	**0.010**	0.428

Tabla S2. Level of significance of the different Postharvest related factors and their interactions on the consumer mandarin choice. The total number of participants in the conjoint test was 280. *Cd:condition.*

POSHARVEST FACTORS	
Attributes	
Waxing	**< 0.0001**
Rind Condition	**0.000**
Calyx Condition	**0.007**
Waxing * Calyx Cd	**0.000**
Waxing * Rind Cd	0.242
Calyx Cd * Rind Cd	**0.020**
